# Revisiting critical digital health studies in the age of generative AI: Research in *Health* and beyond

**DOI:** 10.1177/13634593261451272

**Published:** 2026-05-26

**Authors:** Deborah Lupton

**Affiliations:** 1 Vitalities Lab, Centre for Social Research in Health and Social Policy Research Centre, University of New South Wales (UNSW), Sydney, Australia

**Keywords:** environment and health, experiencing illness and narratives, social inequalities in health, technology in healthcare

## Abstract

This review article for *Health*’s 30th anniversary special issue provides an update on an article I contributed for the 20th anniversary of *Health* in 2016 which outlined how the sub-field of critical digital health studies has been explored in the journal. The current review article is in two parts. It begins with an overview of digital health research published in *Health* in the decade since the first review was published, reflecting on the methods and conceptual approaches that have been used over the past 10 years to engage with this topic. The second part goes on to consider how critical digital health studies might address the latest ‘big thing’ in digital health: generative AI applications. Some bold claims have been made about the benefits that generative AI models are supposed to offer patients and healthcare providers, but their impacts have not yet been fully evaluated from a critical digital health perspective. The article ends by emphasising the importance of acknowledging and identifying how the introduction of generative AI applications into medicine and health care can harm humans and the ecosystems of which they are part. Critical digital health studies should therefore expand its focus beyond its current human-centric preoccupations. As an interdisciplinary social research journal from its inception, *Health* is well-placed to offer a home for such critical analyses.

## Introduction

This review article for *Health*’s 30th anniversary special issue provides an update on an article I contributed for the 20th anniversary of *Health* which outlined how the sub-field of critical digital health studies had been explored in the journal (Lupton, 2016b). My definition of critical digital health studies incorporated social, cultural and political analysis of how digital health technologies were experienced by lay people and healthcare professionals, including analysis of the health information that was created and shared in online media, and the implications for understandings of health, illness, healthcare and medical expertise.

In my review, I remarked on the profound impacts of the internet, digitalisation and mobile computing devices on the ways that lay people learned about health issues and shared their knowledge with each other through online forums, discussion boards, YouTube and the social media platforms, including Facebook, Twitter and Instagram, that had emerged in the intervening two decades. I made mention of the proliferation of dedicated patient support platforms such as PatientsLikeMe, big data analytics about health and illness, virtual environments for medical training, mobile apps related to health, ‘smart’ devices that were connecting to each other in what was referred to as ‘the Internet of Things’, and the emergence of wearable devices such as fitness monitors and smartwatches that facilitated self-tracking of biometrics, assisting people to generate their own health data.

I went on to provide an overview of *Health* articles (appearing from 2000–2015) that had been published investigating these topics. My review ended with proposing new directions for critical digital health studies, including examining the proliferation of ways in which human bodies have become datafied and the implications for health data privacy and security. I suggested that sociomaterialism perspectives offer a fruitful way forward for theorising and empirically investigating the impacts of these dynamic phenomena on human embodiment, subjectivity and social relations.

The 20th anniversary article on critical digital health studies introduced what in 2016 was a new area of research. The current review for the 30th anniversary is divided into two parts. First, I provide an overview of digital health research published in *Health* in the decade since my first review was published (from January 2016 to December 2025, including those that were published online first). I reflect on the methods and conceptual approaches that have been applied over the past 10 years to engage with this topic. The second part of this article goes on to consider how critical digital health studies might address the latest ‘big thing’ in digital health: generative AI applications. The article ends in a similar way to my 20th anniversary piece – by noting gaps in the critical digital studies literature and suggesting what further research should be conducted in the decade to come, particularly in relation to generative AI.

Generative AI is the term used to refer to recent developments in artificial intelligence (AI) tools involving large language models and image generator models trained on massive data sets, mostly collected from the internet, to identify patterns that are then used to generate new text, code, audio or visual content. The most well-known examples of generative AI applications are OpenAI’s ChatGPT, Microsoft’s Copilot, Google Gemini and Claude by Anthropic, together with the image-generating services DALL-E and Midjourney. The human-like conversational style used in the content created in response to users’ queries as well as these applications’ ability to create written text and realistic visual and audio materials have led to significant uptake across the world (Chatterji et al., 2025). These technologies have received intense publicity and hype since late 2022, when OpenAI released ChatGPT. Other Big AI Tech corporations such as Meta, Google, Amazon, Microsoft, Anthropic and xAI are directing significant resources towards developing and promoting generative AI, building massive data centres and other infrastructure to support it and inserting these tools into as many domains of social and economic life as they can (Brennan et al., 2025; Hanna and Bender, 2024).

In relation to medicine and healthcare, some bold claims have been made about the benefits that generative AI technologies offer patients and providers. As I show in this article, their impacts have not yet been fully evaluated from a critical digital health perspective.

Since the 20th anniversary issue was published in January 2016, there has been a steady stream of articles focussing on the application of a range of digital technologies to health and medical care published in *Health*. Across this latest decade, a total of 55 digital health articles were published. Table 1 provides an overview of the topics that have been covered and the conceptual approaches employed.

**Table 1.** Articles about digital health published in Health, January 2016–December 2025.

Year	Topic and technology	Conceptual approach	Authors
2016	Critical digital health studies (range of digital technologies)	Critical perspectives, medical power, lay expertise	Lupton (2016b)
	Online health information sharing (websites, listservs, social media)	Public illness experiences, peer connexion	Conrad et al. (2016)
	Online narratives of eating disorders on a website	Illness narratives, care and control	Hipple Walters et al. (2016)
	Men’s experiences of infertility on a discussion board	Emotional aspects of health experiences, peer connexion	Hanna and Gough (2016)
	Popular nutrition bloggers	Epistemic expertise, rhetoric	Huovila and Saikkonen (2016)
	Physical activity self-monitoring (computer support package)	Sociomaterialism, disciplinary practices	Lynch and Cohn (2016)
2017	Reducing alcohol consumption (website and personal blog)	Online health promotion and self-expression	Carah et al. (2017)
	Self-tracking health conditions (digital monitors)	Subversive micropolitics, sociomaterialism	Fox (2017)
	Weight-loss websites	Theories of practice, self-quantification	Niva (2017)
	Portrayals of fat bodies in digital news reporting	News framing, weight stigma	Atanasova and Koteyko (2017)
2018	Mental health support forum for adolescents	Interaction, conversational analysis	Kaufman and Whitehead (2018)
	Discussion forums on ADHD medication and aspartame	Discursive psychology, interaction	Versteeg et al. (2018)
2019	Use of digital media to connect to health care (range of digital technologies)	Socio-ethical issues, risk, regulation	Petersen et al. (2019b)
	Patient disempowerment due to commercial access to digital health records	Power relations, bioethics	Ebeling (2019)
	Digital risk calculators (websites)	Bioethics, risk theory	Skolbekken (2019)
	Corporate wellness and digital self-tracking (apps, wearable devices, wellness programmes)	Attention economy, active subjects, libidinal energies	Till (2019)
	Fundraising campaigns for stem cell treatments (crowdfunding websites)	Politics of evidence, illness narratives	Tanner et al. (2019)
	Blog discussions about treatment of bipolar disorder	Lay expertise, tactics of everyday practice	Egher (2019)
	Digital media for patient activism (news sites, websites, campaign materials)	Bio-digital citizenship, lay expertise	Petersen et al. (2019a)
	Social media engagement with a food infotainment TV show (Facebook and Twitter)	Audience research, content analysis, health communication	Declercq et al. (2019)
	Online self-tracking of daily activities on the Blipfoto photo-sharing platform	Practice theory, models of self-care	Brewster and Cox (2019)
2020	Diabetes patients sharing support and information on Facebook	Lay expertise, bio sociality online, praxiography	Kingod (2020)
	Fitness self-tracking using the Fitbit wearable device	Foucauldian and sociomaterialism theory	Esmonde and Jette (2020)
	Mothers’ discussions of highly sensitive children on a parenting discussion forum	Intensive mothering, parenting norms, rhetorical resources	Edenroth-Cato (2020)
	ADHD information in the media (websites, news articles, TV programmes)	Discourse analysis, health communication	Ponnou et al. (2020)
2021	Surgical teams’ use of robotic-assisted surgery	Division of labour in healthcare, negotiated order, realist enquiry	Randell et al. (2021)
	Collecting patient feedback using digital screens in medical clinics	Health policy, sensory experience, multimodality	Ong and Sanders (2021)
	Telehealth services, informational medicine and the doctor-patient relationship	Doctor-patient interaction, medical expertise, dehumanisation	Botrugno (2021)
	Social media use among young adults with long-term conditions	Online health communities, peer support	Wilson and Stock (2021)
	News stories about cancer on Facebook	Framing analysis, health communication	Starr and Oxlad (2021)
	Crowdfunding platforms for Lyme disease patients	Contested illness, illness narratives	Vassell et al. (2021)
2022	Birth narratives on a parenting forum	Narrative analysis, maternal embodiment and subjectivity	MacLellan (2022)
	Digital correspondence to a food agency from lay people (web forms or email)	Critical discourse analysis, health communication	Bergman et al. (2022)
	Contested illness narratives on YouTube and Instagram	Moral legitimacy, lay expertise, self-care	Groenevelt (2022)
2023	Health awareness on Fitbit and ParticipACTION websites	Rhetoric analysis, the optimised self, moral meanings	Gaudet (2023)
	Algorithms in automated clinical decision-making	Algorithmic authority, logics of care, medical work	Torenholt and Langstrup (2023)
	Telehealth services and digital divides	Social determinants of health, health policy	Warr et al. (2023)
	Promissory discourses related to a COVID contract tracing app	Content and discourse analysis, sociology of expectations	Samuel and Sims (2023)
	Digital health data economy and the welfare state	Commodification of health data, solidarity	Snell et al. (2023)
	Telemedicine services for people in COVID-19 quarantine facilities	Technogeography of care, heterotopia	Bar-Lev (2023)
2024	Digital self-monitoring for multiple sclerosis (activity tracker and app)	Sociomaterialism, enactment of self-care, lifeworld’s	Wendrich and Krabbenborg (2024)
	Teletherapy and spiritual caregiving (messaging apps, video calls and conferencing platforms)	Intercorporeality, sensory studies	Popper-Giveon and Keshet (2024)
	Digital social alarm systems for older people	Sociomaterialism, enactment of care	Lydahl (2024)
	Self-tracking young people’s engagement with body ideals through social media (Facebook, Instagram, TikTok, Snapchat, YouTube)	Body image, emotion	Stage and Bach Nielsen (2024)
	Avoidance of self-tracking for menopause (‘un-tracking’)	Post-phenomenology, situated bodily awareness	de Boer et al. (2024)
	Digital treatment and rehabilitation for back pain (bespoke platform)	Temporal agency, patient empowerment	Christiansen et al. (2024)
2025	Teletherapy (video calls and conferencing platforms)	Vital materialism, more-than-human care, assemblage theory	Kolehmainen (2025)
	Patient-centred telemedicine in primary care	Digitised care, medical work	Keshet et al. (2025)
	Trial of a teleoperated care robot in a hospital ward	Feminist posthumanism, enactment of care	Nettleton et al. (2025)
	Video consultation in general practice	Enactment and norms of care	Lüchau et al. (2025)
	Online booking system for COVID-19 vaccine appointments	Social inequalities in healthcare access	Ghomari et al. (2025)
	Telemedicine used by primary care physicians for communication (phone, email services)	Boundary work, boundary objects, medical expertise	Popper-Giveon et al. (2025)
	Femtech (range of devices and services)	Feminist new materialism, diffractive analysis across disciplinary perspectives	Facca et al. (2026)
	Communication on an eHealth platform	Affective atmospheres, communication gaps	Mahnke (2026)
	Machine learning decision support systems for healthcare professionals	Biomedical ethics, science and technology studies, sociomaterialism	Willem et al. (2025)

As this table shows, the number of articles about digital health published each year including and from 2016 have ranged from only two in 2018 to a high of nine in the following year (six of which were part of a special issue on the socio-ethical and regulatory implications of lay people’s use of digital media to connect with health care) and in the latest year of 2025. In most other years, 4–6 articles on digital health were published.

In my 2016 review, I noted that *Health* articles predominantly focussed on analysing patient-generated contributions to online forums and online health information-seeking behaviour. I observed that there had been very little analysis published in the journal on healthcare providers’ adoption of digital technologies or patients’ use of apps, social media, telehealth services and at-home digital monitoring technologies. It is striking that little change has occurred since then, with most analyses across the most recent decade addressing online health information or patients sharing their experiences on health websites, blogs or online discussion forums. Only a minority of more recent articles have focussed on health information shared on social media platforms. Only one (briefly) refers to TikTok, the social media app that had become particularly popular among young people by 2019, and which has received sustained attention elsewhere for its content on health topics (Ramsden and Talbot, 2025).

However, several changes over time in topics addressed by digital health researchers are evident in the collection of *Health* articles published since 2016. The early years of this century were characterised by increased medical and social science attention to fat embodiment and the ‘obesity epidemic’ (Lupton, 2018; Wright and Gard, 2005). This is reflected in several articles on this topic in *Health*’s coverage of digital health topics. By the middle of the second decade of this century, the phenomenon of digital self-tracking became a popular topic in digital sociology, digital health and media and communication studies (Lupton, 2016a; Neff and Nafus, 2016). This topic has also been well covered in *Health* since 2016, including several recent articles. Most notably, the outbreak of the COVID-19 pandemic in early 2020 and with it, a turn in many healthcare systems towards offering telemedicine and remote health monitoring services during periods of stay-at-home orders in the pandemic’s emergency phase, has resulted in a renewed interest from contributors to the journal in how patients and healthcare providers are engaging with such services.

Since 2016, methods adopted by researchers exploring digital health topics include discourse, conversation and rhetoric analysis of content shared on platforms and social media. Other well-used approaches involve analysis of the illness experience, power relations, medical and lay expertise and doctor-patient relationships as they are mediated with and through digital technologies. Identifying concepts of embodiment, care and self-discipline in digital health use have also been popular for *Health* contributors over the past decade. All these approaches were evident in the decade prior to 2016 (Lupton, 2016b).

In my 2016 review, I suggested that sociomaterialism perspectives had much to offer for critical digital health studies. Since then, sociomaterialism has received greater attention in *Health* across different topics, particularly in the last few years. So too, as shown in Table 1, several *Health* articles published since 2016 have adopted a sociomaterialism lens in relation to digital health, investigating how humans enact care or health states with and through objects such as eHealth, health monitoring or automated decision-making systems, and mobile apps and devices. What is not so evident in *Health* is scholarship seeking to investigate the entanglements of human embodiment and health states with other living things and ecosystems. It is also important to draw attention to the fact that the focus of digital health analyses in *Health* since 2016 has predominantly been on populations or sub-groups living in wealthy Western nations, an issue I pointed out at the time. Ten years later, the continuing lack of attention to the Majority World and other marginalised social groups is a stark reminder that consideration of how other populations engage with the ‘digital health transformation’ (Kickbusch et al., 2021) is frequently lacking. This trend is driven by a global health colonial mindset and continuing inequities related to how research is funded and who is able to conduct and publish it (Osmanlliu et al., 2025).

Together, these absences signal a continuing human-centric and Global North-focussed orientation in *Health*’s critical digital health publications to date. Even those authors using sociomaterial analyses tend to highlight the role played by technological and other non-living agents in digital health encounters rather the enactments of humans with nonhuman living beings or with features of landscapes such as air, soil and water. Incorporating these more-than-human perspectives into critical digital health studies is crucial as people and the planet face the impacts of the rapid expansion of generative AI infrastructures.

## Tackling the generative AI phenomenon

Up until December 2025, *Health* had not yet published any articles on the generative AI boom. This is perhaps unsurprising, given the novelty of these applications and the time required to conduct empirical social research. Yet there is now an extensive body of work published in medical, public health and computer science journals about the potential of generative AI to contribute to the fields of medicine and public health. In these forums, generative AI applications are often described as having ‘transformative’ potential to ‘revolutionise’ healthcare, health promotion and public health systems, and deliver ‘precision’ or ‘personalised’ medicine.

As scoping reviews have shown (Akbarialiabad et al., 2025; Rashidi et al., 2025), it has been suggested that these tools can assist with diagnosis, screening, clinical decision-making, knowledge retrieval, medical note-taking and recordkeeping, medication management, triaging patients, clinical data and workflow management and as virtual assistants for telemedicine. Some claims have been made that generative AI applications could be used in medical and allied healthcare education, training and research. AI-assisted ‘wellness’ apps have been developed as a way of contributing to health promotion efforts. These include specialised ChatGPT or Gemini apps designed to help people with mental health difficulties by using chatbots as therapists (De Freitas and Cohen, 2025) or offering ‘personalised feedback’ to users on lifestyle aspects such as fitness training, sleep patterns, weight loss and stress levels to help people improve their health and wellbeing (Lederman et al., 2025).

However, many criticisms have also been made of generative AI systems’ use in health and medicine contexts. These applications are well-known for the persistent errors (or ‘hallucinations’) in the content they create, including in published scientific literature (Naddaff and Quill, 2026). People are using these services to self-diagnose and receive advice about health problems (Costa-Gomes et al., 2026) but their value remains unclear. When deployed in a clinical setting, one of the main criticisms of generative AI is its potential for inaccuracy, lack of comprehensiveness in diagnoses and propensity to give only vague answers (Hasan et al., 2025). A study testing whether large language models can assist members of the public in identifying underlying conditions and choosing a course of action in 10 medical scenarios found that in many cases, participants were not able to use them effectively or safely. The process therefore did not successfully follow a typical doctor-patient encounter, where experienced clinicians can assess the context of each interaction, probe patients for further information or clarification if required (Bean et al., 2026).

As automated systems using large language models to statistically process language, chatbots are incapable of providing compassionate, authentic care. They are deceptively fluent and articulate in terms of their conversational communication style and are often promoted as ‘friends’ or ‘companions’ (Guersenzvaig and Delgado, 2025). However, these services are prone to making useless generic statements or giving the wrong advice to patients (Bulian et al., 2024). By contrast with AI, human healthcare providers’ ability to sense, understand, respond and empathise with patients dealing with complex health difficulties can be a crucial benefit (Forsyth et al., 2024). Of particular concern is that inappropriate and even harmful advice can be given to vulnerable patients such as those considering self-harm, given the propensity for app developers to present their chatbots as trustworthy human-like interlocuters (De Freitas and Cohen, 2025).

Bias involving gender, race, religion, and political orientation is also evident in the responses generated by these tools, due to embedded biases in the data on which they are trained (Motoki et al., 2024). In the medical arena, these risks include perpetuating racial and gender biases which can then lead to discrimination against patients by healthcare professionals and incorrect medical diagnoses (Zack et al., 2024). Researchers have also drawn attention to the multiple ways that the introduction of these applications into healthcare can lead to the deskilling and loss of autonomy for clinicians and other healthcare professionals. Deferring skills to AI models such as consultation, diagnosis and the provision of information to patients reduces the human contact and decision-making that healthcare providers rely on to provide sensitive and effective patient care (Natali et al., 2025).

Further, some questions around generative AI applications have emerged regarding patient information privacy and security harms. It is also problematic that many of these AI healthcare initiatives are driven by Big Tech companies who are interested in maximising profit over safety and privacy protections, and that governments are not stepping in to ensure appropriate regulations and standards are met (Zaim and Shaw, 2026). Medical researchers have therefore called for greater regulatory oversight of these models and the development of robust policy frameworks to better protect against these risks (Hasan et al., 2025).

Many of these issues remain speculative, as to date, little social research has been published on how people, including patients and healthcare providers, are engaging with generative AI in health settings. There are many potential directions for critical, social theory-driven approaches that investigate health care professionals’ and lay people’s lived experiences of generative AI and what they would like to see in further developments and iterations so that these applications can improve healthcare rather than detracting from its quality or causing harm. Investigating the meanings of using these applications for health and medical purposes remains important, particularly addressing the cultural specificities of such use. Any technological artefact is an object imbued with meaning and narrative (Lupton, 2014, 2016b). The story-telling and descriptive language used to promote technologies and make claims for their benefits is a crucial element of how they are understood by target users. This is the case of generative AI for health just as it is for other digital technologies such as mobile apps, fitness trackers, health monitors or care robots (De Matas, 2025; Guersenzvaig and Delgado, 2025; Lupton, 2016a).

Another area that requires a critical digital health approach based on investigations into lived experience is the issue of errors and potential misinformation spread through generative AI systems. Although the consensus in the medical and public health literature is that this is a huge concern with generative AI services (Bandeira et al., 2025; Costa-Gomes et al., 2026), there is a lack of empirical research that investigates the volume, content, and ‘uptake’ of this misinformation. A major question remains concerning to what extent both clinicians and patients can invest their trust in generative AI. Until this is possible, most applications in the medical and health domain will be limited.

Social researchers can bring in-depth methods together with social theory to investigate how trust operates in these settings and to what extent and under what conditions generative AI applications are accepted in these environments. They can explore the hidden labour required by both patients and healthcare providers when using these applications. Researchers can probe into issues such as whether ChatGPT and other large language models are having an actual effect on the amount of misinformation that people consume, and if so, what is the nature of that misinformation and what kind of impact it is having on people’s health outcomes, clinical decisions and public health at a broader level. Another avenue to be pursued is investigating whether there is a meaningful difference in how generative AI services are shaping avenues for health-related misinformation, versus a common internet search, and how lay people evaluate the accuracy of the responses they receive from their health-related queries to large language models.

A further topic for social inquiry is identifying the reasons why chatbots might be appealing to a patient compared with going to a clinician (e.g. social structural and economic factors such as cost and accessibility of health services). As noted earlier, critical digital health studies approaches, in *Health* and elsewhere, have frequently pointed out how power imbalances in the doctor-patient relationship and neoliberal political ideologies concerning personal responsibility for achieving good health have been reproduced in digital health applications. The ways that generative AI technologies contribute to these issues also require further investigation.

Beyond generative AI technologies’ direct use for medical applications, there is another set of serious issues worthy of exploration that relate to the broader social structural and political dimensions of their deployment. Scholars in critical AI and data studies have pointed to the extractivist, racist, imperialist and exploitative ethos motivating Big AI Tech in their quest for profit (Brennan et al., 2025; Hanna and Bender, 2024; Mejias and Couldry, 2024; Tacheva and Ramasubramanian, 2023). These corporations’ practices of seeking to expand generative AI use and build the infrastructures required to support this expansion are already having huge impacts on humans and the natural world. The generative AI boom is accompanied by the expansion of massive infrastructural components to support it, including data centres and marine cabling (Hogan, 2024; Wang et al., 2024). These infrastructures expose humans and other elements of ecosystems to significant habitat disruption and environmental hazards from land-clearing, deep sea tunnelling, greenhouse gas emissions and impacts caused by its heavy energy use (de Vries-Gao, 2026; Lupton, 2025; Osmanlliu et al., 2025). In 2025, it was calculated that the hyperscale data centres built and run by Big AI Tech were already consuming 4.4% of the US energy supply (O’Donnell and Crownhart, 2025). Water resources are consumed in large quantities by data centres: including in regions where drought is a problem, threatening the health of the local environment as well as food production (Mccauley and Scanlan, 2025).

The mining of rare earth minerals used in manufacturing the computer chips and other electronic components of generative AI technologies is carried out in countries such as Myanmar and the Democratic Republic of Congo under terrible working conditions, exposing people, including children, to human rights abuses and significant harm of injury or exposure to toxic chemicals (Brown et al., 2024; Lungu, 2025). The expansion of generative AI infrastructures also leads to greater e-waste (discarded electronic devices, batteries, circuit boards, chips and other infrastructural components). The disposal, management and processing of e-waste is disproportionately located in the regions in the Majority World such as Ghana, Nigeria, China, Brazil, India, the Philippines and Pakistan, exposing populations living nearby or working on e-waste recycling to major hazards from air, water and soil pollution due to the spilling or leakage of toxic chemicals and heavy metals (Bhardwaj et al., 2025; Sandwal et al., 2025).

Another way in which generative AI systems and infrastructures pose considerable harms to human health is through exploitation of workers working on training or moderating digital data content. These digital workers are the invisible labour force that fine-tune and check the models’ data input and output, supporting the claims of Big AI Tech that large language models possess human-like intelligence (Ruggiu and Özdemir, 2026). Typically these digital workers provide outsourced labour for wealthy companies such as OpenAI and Meta in countries in Africa, Latin America and Asia, with few rights and poor working conditions (Ruggiu and Özdemir, 2026; Tubaro et al., 2025). Researchers have identified the psychological effects on AI data workers in the Majority World who are tasked with helping train large language models to detect and filter toxic content (Mejias and Couldry, 2024; Tacheva and Ramasubramanian, 2023).

The possible impacts of the latest AI systems on health therefore go well beyond potential clinical applications and into areas such as the health harms caused by exploited labour and the environmental impacts of these technologies on humans and the environment. Indeed, given their potential to undermine human and planetary health, some scholars have argued that generative AI technologies should be considered part of the digital determinants of health. By extending this perspective on the extractivist and exploitative ethos of Big Tech to how these activities affect the natural world, the notion of digital planetary health can offer some insights (Ip, 2024; Lupton, 2025). There is a pressing need for focussing generative AI research on settings outside the Global North (Wakunuma and Eke, 2024). In most research on generative AI and health, there is evidence of embedded narrative logics about whose experiences count and whose futures are imagined. Furthermore, the logics of medicine and care that underpin generative AI health applications are framed through a Western perspective that is often not relevant or appropriate to the worldviews and practices of people in the Majority World (De Matas, 2025). A review of research contributions to the published literature about generative AI in public health found that US-based authors dominated by far, with one prestigious university in that country leading the way (Favara et al., 2024). This literature presents a solid foundation for conducting further critical scholarship into the political and socioeconomic dimensions of the impacts of generative AI boom.

## Concluding comments: Future directions in critical digital health studies

In my 20th anniversary review, I called for more critical and in-depth analyses of how people use digital health devices and software; the ways that digital data are visualised and materialised; how people make sense of and act on their personal health data; and the political and privacy issues around how health data are used by third parties. As I have shown in this 30th anniversary review, since then, *Health* contributors have addressed all these topics to some extent. However, I argue that what has been lacking thus far is a planetary digital health perspective, emphasising the importance of acknowledging and identifying how the introduction of generative AI technologies into medicine and health care can harm humans and the ecosystems of which they are part. In a current global context in which planetary health is ever more threatened by crises such as climate change, pollution and loss of biodiversity, to which the rapid expansion of generative AI technologies and infrastructures is contributing, critical digital health studies should expand its focus beyond its current human-centric preoccupations. I therefore invite digital health researchers contributing to *Health* to consider adopting a more-than-human perspective that considers how the health of all elements of ecosystems, human and nonhuman, are affected, for better or for worse, by digital technologies.

This review also demonstrates that there needs to be greater diversity in the geographical location and cultural contexts of research on these topics, so that the benefits, barriers and risks that are specific to less privileged populations are better understood. Therefore, I further call for a decolonial approach in critical digital health studies that is inclusive of Majority World populations and led by researchers from those regions. The concept of digital colonialism (Kakar, 2021; Mwema and Birhane, 2024) should be extended to incorporate health and medical perspectives. As De Matas (2025, emphasis in the original) states, ‘health AI systems must be constructed *with* communities, not merely delivered *to* them’. Situated ethical consideration of these technologies must involve epistemic inclusion across cultural contexts, allowing lay people and healthcare providers from different backgrounds to have a say in evaluating their benefits and risks using their own worldviews rather than those imposed upon them. As an interdisciplinary social research journal from its inception, *Health* is well-placed to offer a home for such critical analyses.
